# Cryptic speciation of the zoogonid digenean *Diphterostomum flavum* n. sp. demonstrated by morphological and molecular data

**DOI:** 10.1051/parasite/2020040

**Published:** 2020-06-18

**Authors:** Carmen Gilardoni, Jorge Etchegoin, Thomas Cribb, Susana Pina, Pedro Rodrigues, María Emilia Diez, Florencia Cremonte

**Affiliations:** 1 Laboratorio de Parasitología, Instituto de Biología de Organismos Marinos (CCT CONICET-CENPAT) Boulevard Brown 2915 U9120ACF Puerto Madryn Argentina; 2 Laboratorio de Parasitología, IIPROSAM – Instituto de Investigaciones en Producción Sanidad y Ambiente, FCEyN, Universidad Nacional de Mar del Plata – CONICET Juan B. Justo 2550 7600 Mar del Plata Argentina; 3 School of Biological Sciences, The University of Queensland Brisbane 4072 Queensland Australia; 4 Laboratorio de Sanidade, ICBAS – Instituto de Ciencias Biomédicas Abel Salazar, Universidade do Porto R. Jorge de Viterbo Ferreira 228 4050-313 Porto Portugal; 5 Laboratorio de Imunidade Inata e Ferro, I3S – Instituto de Investigação e Inovação em Saúde, Universidade do Porto R. Alfredo Allen 4200-135 Porto Portugal

**Keywords:** Life cycle, Zoogonidae, *Pinguipes brasilianus*, Cryptic species, Patagonian coast

## Abstract

*Diphterostomum brusinae* (Zoogonidae) is a digenean species that has been recorded worldwide parasitizing marine fishes. Several species have been synonymized with *D. brusinae* because they lack conspicuous morphological differences. However, due to the breadth of its geographic distribution and the variety of hosts involved in the life cycles, it is likely to be an assemblage of cryptic species. *Diphterostomum flavum* n. sp. is described here as a morphologically cryptic relative of *D. brusinae*, in the fish *Pinguipes brasilianus* (Pinguipedidae) off the Patagonian coast, Southwestern Atlantic Ocean, and its life cycle is elucidated through morphology and molecular analysis. This species uses the gastropod *Buccinanops deformis* (Nassariidae) as first and second intermediate host with metacercariae encysting within sporocysts. They also, however, use the polychaete *Kinbergonuphis dorsalis* (Onuphidae) as second intermediate host. No morphological differences were found between adults of *D. flavum* n. sp. and *D. brusinae;* however, the number of penetration glands of the cercariae, a diagnostic feature, differed (9 vs. 3 pairs), as well as the ITS2 sequences for the two species. This work provides morphological and molecular evidence of cryptic diversification among species described as *D. brusinae*, in which the only clear differences are in larval morphology and host spectrum. The strict specificity to the snail acting as the first intermediate host and the variety of fishes with different feeding habits acting as definitive hosts support the likely existence of multiple cryptic species around the world.

## Abstract (fr)

*Diphterostomum brusinae* (Zoogonidae) est une espèce de digène, parasite des poissons marins, qui a été signalée dans le monde entier. Plusieurs espèces ont été synonymisées avec *D. brusinae* parce qu’elles ne montrent pas de différences morphologiques visibles. Cependant, en raison de l’étendue de sa répartition géographique et de la variété d’hôtes impliqués dans les cycles de vie, il est probable qu’il s’agisse d’un assemblage d’espèces cryptiques. *Diphterostomum flavum* n. sp. est décrit ici comme une espèce morphologiquement cryptique de *D. brusinae*, chez le poisson *Pinguipes brasilianus* (Pinguipedidae) au large de la côte de la Patagonie, dans le sud-ouest de l’océan Atlantique, et son cycle de vie est élucidé par la morphologie et l’analyse moléculaire. Cette espèce utilise le gastéropode *Buccinanops deformis* (Nassariidae) comme premier et deuxième hôte intermédiaire avec des métacercaires s’enkystant dans les sporocystes. Cependant, elle utilise également le polychète *Kinbergonuphis dorsalis* (Onuphidae) comme deuxième hôte intermédiaire. Aucune différence morphologique n’a été trouvée entre les adultes de *D. flavum* n. sp. et *D. brusinae*, mais le nombre de glandes de pénétration des cercaires, une caractéristique diagnostique, diffère (9 paires contre 3), ainsi que les séquences ITS2 pour les deux espèces. Ce travail fournit des preuves morphologiques et moléculaires de la diversification cryptique parmi les espèces décrites comme *D. brusinae*, dans lesquelles les seules différences claires sont dans la morphologie des larves et le spectre de l’hôte. La spécificité stricte au mollusque servant de premier hôte intermédiaire et la variété de poissons avec différentes habitudes alimentaires servant d’hôtes définitifs soutiennent l’existence probable de plusieurs espèces cryptiques dans le monde.

## Introduction

**Figure 1:**
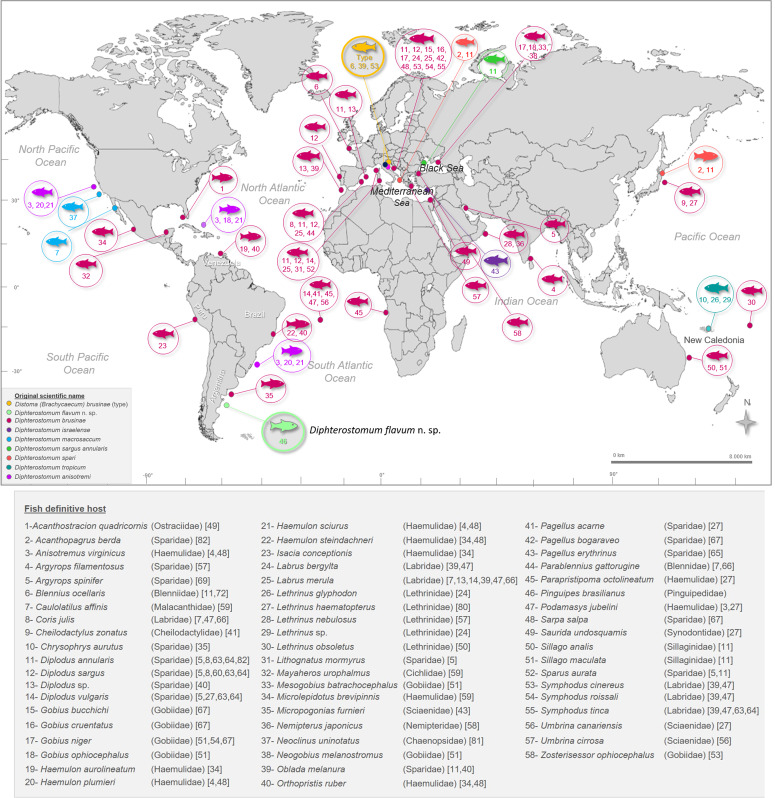
Geographical distribution of species described as *Diphterostomum brusinae* (Stossich, 1888) (Digenea, Zoogonidae) from fish around the world. References for each record are given between brackets.

Cryptic speciation has been reported to be common among parasites. Due to a limited range of morphological features among parasite taxa, many species exhibit similar or identical morphology; however, they may differ in host-parasite interactions [45]. Cryptic species are typically discovered through molecular genetics, behavioral, or ecological studies of diversity [19]. Using molecular data, cryptic diversity has been detected extensively in trematodes species [19, 29, 33, 44]. Digenean species with homogeneous morphology but infecting a wide range of host species are candidates for complexes of cryptic species [29, 37, 46]. This may be the case for *Diphterostomum brusinae* (Stossich 1888), a zoogonid trematode parasitizing the intestine of fish, which has been recorded in North and South America, Europe, Asia, and Oceania (Fig. 1). Molecular data for this species and related genera are scarce; only the sequences of ribosomal DNA (18S and ITS1) for *D. brusinae* from North of Portugal are available [26, 60].

**Figure 2:**
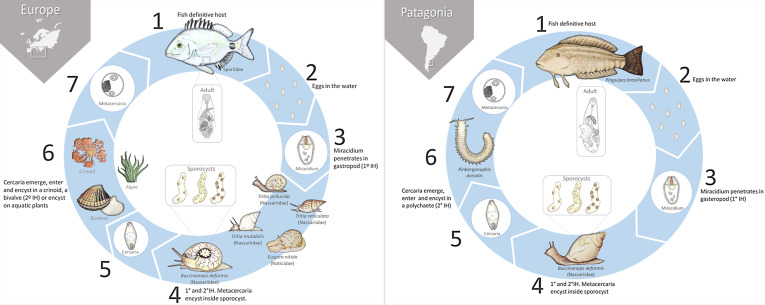
Schematic diagram of the known life cycles of the digenetic zoogonids *Diphterostomum brusinae* from Europe and *Diphterostomum flavum* n. sp. from Patagonia (South America). *Abbreviations*: IH, intermediate host. References: [21, 25, 54, 55, 60, 62].

The life cycle of *D. brusinae* has been elucidated from the Mediterranean Sea [54, 55], Black Sea [21], and the Atlantic coast of Portugal [26, 60] (Fig. 2). In all these studies, gastropods are the first intermediate hosts and metacercariae were found encysting inside the sporocysts. Metacercariae can also develop outside the sporocysts. The list of second intermediate hosts includes gastropods, bivalves and other sedentary invertebrates, such as the crinoid *Antedon mediterranea* [62]. Occasionally, the metacercariae encyst on the surface of algae and aquatic plants [21].

In Argentina, Timi et al. [77] and Gilardoni et al. [31] reported the adult of *D. brusinae* in the Brazilian sandperch *Pinguipes brasilianus* Cuvier (Pinguipedidae), from the coasts of Buenos Aires Province, San Matias Gulf, and Nuevo Gulf (Patagonia). Later, Martorelli et al. [43] reported it from the Whitemouth croaker, *Micropogonias furnieri* (Desmarest) (Sciaenidae), from the Bahía Blanca estuary and Samborombón Bay (Buenos Aires province). With respect to intra-molluscan stages, sporocysts (containing cercariae and/or metacercariae) of a *Diphterostomum* species were found parasitizing the intertidal snail *Buccinanops deformis* (Kiener) (Nassariidae) in Nuevo Gulf [31], and in Samborombón Bay [43].

Here we described *D. flavum* n. sp. from *Pinguipes brasilianus* from off the Patagonian coast, Argentina, elucidated its life cycle, and identified it as a cryptic species of *D. brusinae* using morphological and molecular data.

## Materials and methods

### Data collection and parasite study

Intra-molluscan stages (sporocysts, cercariae, and intra-sporocyst metacercariae) of *Diphterostomum* sp. were previously described by Gilardoni et al. [31] from the gastropod *Buccinanops deformis* from the intertidal and shallow subtidal regions of Punta Cuevas (42°46′ S, 64°54′ W), Puerto Madryn, Chubut Province, Argentina. To improve the prevalence data, *B. deformis* were sampled from two additional intertidal sites (Table 1). Gastropods were dissected alive and the presence of parasite (sporocysts) was recorded.

**Table 1:** List of invertebrates and fish dissected for developmental stages of *Diphterostomum flavum* n. sp. from the Patagonian coast, Argentina. Localities: FB, Fracasso Beach (San José Gulf) (42°25′43″ S, 64°07′46″ W); CB, Cracker Bay (Nuevo Gulf) (42°51′33″ S, 64°46′16″ W); PE, Punta Este (Nuevo Gulf) (42°47′13″ S, 64°56′49″ W); PM, Puerto Madryn (Nuevo Gulf) (42°46′ S, 64°54′ W); PL, Puerto Lobos (San Matías Gulf) (41°58′50″ S, 65°04′05″ W).

Host examined	Locality	Collection date	N examined	Prevalence (%)	Mean intensity	Stages	Infection site
*Buccinanops deformis* King (Gastropoda: Nassariidae)	FB	May–Jan 2011	430 (I)	3	nd	Sp, C, M	G, DG
	CB	Jan 2014	143 (I)	13	nd	Sp, C, M	G, DG
*Kinbergonuphis dorsalis* (Ehlers) (Polychaeta: Onuphidae)	CB	Jan 2014	72 (I)	22	2 (1–3)	M	Mu
*Ardeamya petitiana* (d’Orbigny) (Bivalvia: Tellinidae)	PM	Oct 2013	50 (I)	–	–	–	–
			13 (S)				
	CB	Jan 2014	30 (S)				
*Ovalipes trimaculatus* (De Haan) (Decapoda: Ovalipidae)	PM	Oct 2013	6 (S)	–	–	–	–
*Boccardia proboscidea* Hartman (Polychaeta: Spionidae)	PM	2010–2013	2666 (I)	–	–	–	–
*Dipolydora* cf*. flava* (Claparède) (Polychaeta: Spionidae)	PM	2010–2013	226 (I)	–	–	–	–
*Spio quadrisetosa* Blake (Polychaeta: Spionidae)	PM	2010–2013	30 (I)	–	–	–	–
*Rhynchospio glutaea* (Ehlers) (Polychaeta: Spionidae)	PM	2010–2013	25 (I)	–	–	–	–
*Boccardiella ligerica* Ferronnière (Polychaeta: Spionidae)	PM	2010–2013	5 (I)	–	–	–	–
*Clymenella* sp. (Polychaeta: Maldanidae)	PM	2010–2013	50 (I)	–	–	–	–
*Cirratulus* sp. (Polychaeta: Cirratulidae)	PM	2010–2013	10 (I)	–	–	–	–
*Halosydna patagonica* Kinberg (Polychaeta: Polynoidae)	PM	2010–2013	5 (I)	–	–	–	–
*Platynereis* sp. (Polychaeta: Nereididae)	PM	2010–2013	25 (I)	–	–	–	–
*Glycera* sp. (Polychaeta: Glyceridae)	CB	Jan 2014	6 (I)	–	–	–	–
*Pinguipes brasilianus* Cuvier (Actinopterygii, Pinguipedidae)	PL	Nov 2012	6 (S)	33	14 (3–21)	A	In
	CB	Mar 2013	3 (S)	33	19	A	In
	PE	Feb 2014	4 (S)	25	4	A	In

*Abbreviations*: I, intertidal; S, subtidal; Sp, sporocyst; C, cercaria; M, metacercariae; A, adult; G, gonad; DG, digestive gland; Mu, muscle; In, intestine; nd, non determined.

To locate the second intermediate hosts of the cycle, the most common macroinvertebrates that cohabit with the molluscan host (*B*. *deformis*) were examined (Table 1). Among these invertebrates, metacercariae belonging to the family Zoogonidae were found only in the polychaete *Kinbergonuphis dorsalis* (Ehlers) (Onuphidae), collected from the sandy sediment using a shovel and a 1-mm mesh. In the laboratory, 72 specimens of *K*. *dorsalis* were flattened between a slide and a coverslip, and examined for parasites under a light microscope. In addition, 13 specimens of *Pinguipes brasilianus* Cuvier (Pinguipedidae), obtained through spear-fishing from three sites (Table 1), were freshly examined for adult stages of *Diphterostomum*. To this end, stomach and intestines of *P*. *brasilianus* were removed and examined under a light microscope. Both metacercariae and adults were studied *in vivo* using both neutral red and Nile blue stained and unstained specimens. Flukes were killed with heated seawater and were immediately fixed with 10% buffered formalin, preserved in 70% ethanol and stained with Semichon’s acetocarmine or Gomori’s trichrome. After being cleared with methyl salicylate, the adults were mounted in Canada balsam and measured. Descriptions of the encysted and excysted metacercariae and adult were based on several live and fixed and stained specimens (*n* = 15). Drawings were made with the aid of a camera lucida, and the measurements are given in micrometers (μm), followed by the range in parentheses. Some of the fixed specimens were dehydrated and dried by rinsing for a few minutes in hexamethyldisilane. The adults were then gold-coated for observation; the samples were photographed using a JEOL JSM-6460LV scanning electron microscope (SEM).

A total of 24 sporocysts, 10 metacercariae, and 19 adults were stored in 96% ethanol for molecular studies. Prevalence of sporocysts, metacercariae and adults and mean intensity of metacercariae and adults were calculated according to Bush et al. [12] (Table 1). Metacercariae from *K. dorsalis* and adults from *P. brasilianus* were deposited at the Parasitological Collection, IBIOMAR, CCT CONICET Centro Nacional Patagónico (CNP-Par 75, 76), Puerto Madryn, Argentina. All the scientific names are used according to WoRMS [79].

Samples of *B. deformis* (*n* = 143) from Cracker Bay (42°51′33″ S, 64°46′16″ W), Nuevo Gulf, Chubut Province, Argentina, was transported live to the laboratory and placed in small flasks filled with seawater at room temperature (20–23 °C). The gastropods were inspected twice daily under a stereomicroscope for emerged cercariae. Experimental infections were performed by placing a large number of emerged cercariae in small containers with target hosts (four polychaetes *Platynereis* sp. (Nereididae), 20 non-native recent invader *Boccardia proboscidea* Hartman (Spionidae), and 20 clams *Ardeamya petitiana* (d’Orbigny) (Tellinidae). Target hosts were collected from Puerto Madryn, where the search for metacercariae had proven negative (Table 1). The target hosts were examined between a slide and a coverslip at the light microscope for parasites nine days post-exposure. Metacercariae found were measured and compared with naturally obtained metacercariae from *K. dorsalis* through a non-parametric Kruskal–Wallis test [70]. Overall prevalences and mean intensity between experimentally and naturally infected metacercariae were compared through a Logistic and Poisson Regression, respectively [16].

### Molecular analyses

DNA from sporocysts from *B. deformis*, metacercariae from *K. dorsalis*, adults from the intestine of *P. brasilianus*, and metacercariae of *D. brusinae* from *Cerastoderma edule* (Linnaeus) (Cardiidae) were extracted using a Sigma-Aldrich GenElute Mammalian Genomic DNA kit (St. Louis, MO, USA). Polymerase chain reaction (PCR) amplifications were performed in a total volume of 50 μL with an amplification profile consisting of 40 cycles of 30 s at 94 °C, 30 s at 54 °C, 120 s at 72 °C, followed by 10 min at 72 °C for the final extension. The ITS2 region of the rDNA was amplified using a digenean specific primer located at 114 base pairs (bp) from the 3′ end of the 5.8S rDNA (5′ – GCTCGTGTGTCGATGAAGAG – 3′), and a specific primer located at 16 bp from the 5′ end of the 28S rDNA (5′ – AGGCTTCGGTGCTGGGCT – 3′). Amplified PCR products were purified using a Qiagen QIAquick Gel Extraction kit (Valencia, CA, USA) and sequenced (Stabvida, Oeiras, Portugal). ITS2 sequences were submitted to GenBank. Sequences were aligned using MAFFT software (available at http://www.ebi.ac.uk/Tools/msa/mafft/). The ITS1 region, a longer variable sequence, was amplified but the sequencing failed. ITS2 sequences of zoogonids *Diphterostomum* sp. (KJ188134.1), *Zoogonus rubellus* (Olson, 1868) (AJ241804.1), *Zoogonus* sp. (KF358773.1), *Deretrema nahaense* Yamaguti, 1942 (KJ188135.1), *Lecithostaphylus gibsoni* Cribb, Bray & Barker, 1992 (KJ188133.1), *Plectognathotrema kamegaii* Cutmore, Miller, Bray & Cribb, 2014 (KM505036.1) as well as two outgroup species of the family Opecoelidae *Macvicaria obovata* (Molin, 1859) (JQ694149.1) and *Cainocreadium labracis* (Dujardin, 1845) (JQ694148.1) were retrieved from GenBank for molecular and phylogenetic studies. Phylogenetic and molecular evolutionary analyses were inferred by both the neighbor-joining (NJ) method using MEGA6 [76] and by Bayesian inference (BI) using BEAST v1.8.0 [23]. To determine the evolution model that gave the best fit to our dataset, the program jModeltest 2.1.1 [18] was employed, with model selection based on the Akaike information criterion (AIC). Results indicated that the general time reversible model with an estimate of gamma distributed among-site rate variation (GTR + G + I) was the most appropriate. For NJ analyses, nodal support was estimated from 1000 bootstrap re-samplings. The resulting trees were rooted with the outgroup taxon. Distance matrices (*p*-distance model, i.e., the percentage of pairwise character differences with pairwise deletion of gaps) were also calculated with MEGA6.

## Results

### Taxonomic information

Subclass: Digenea Carus, 1863

Family: Zoogonidae Odhner, 1902

Genus: *Diphterostomum* Stossich, 1904

### *Diphterostomum flavum* n. sp.

urn:lsid:zoobank.org:act:68BAB7F2-C39F-4692-8AE2-168636015221

Definitive host: *Pinguipes brasilianus* Cuvier (Actinopterygii, Pinguipedidae).

First intermediate host: *Buccinanops deformis* (Kiener) (Gastropoda, Nassariidae).

Second intermediate hosts: *Buccinanops deformis* (intra-sporocyst), *Kinbergonuphis dorsalis* (Ehlers) (Polychaeta, Onuphidae) (natural), *Platynereis* sp. (Polychaeta, Nereididae), and *Boccardia proboscidea* Hartman (Polychaeta, Spionidae) (experimental).

Type-specimens: Holotype, adult 176 (CNP-Par); paratypes, adult 177 (CNP-Par).

Type-locality: Puerto Lobos (San Matías Gulf) (41°58′50″ S, 65°04′05″ W), Patagonian Coast, Southwestern Atlantic Ocean.

Other localities, overall prevalences, infection intensities, and sites of infection: Table 1.

Other specimens deposited: vouchers, sporocyst 6280–6281 (MLP) and 21–23 (CNP-Par), metacercariae 79 (CNP-Par) and 178 (CNP-Par).

GenBank accession numbers: ITS2 sequence of sporocyst (KF358772), metacercaria (MN233043), adult (KF483875), metacercaria of *D. brusinae* from Portugal (MN263044).

Etymology: The specific name is derived from the Latin “*flavum*” meaning “*yellow*” in reference to the color of live adults.

### Developmental life cycle stages of *Diphterostomum flavum* n. sp.

#### Adult

**Figure 3:**
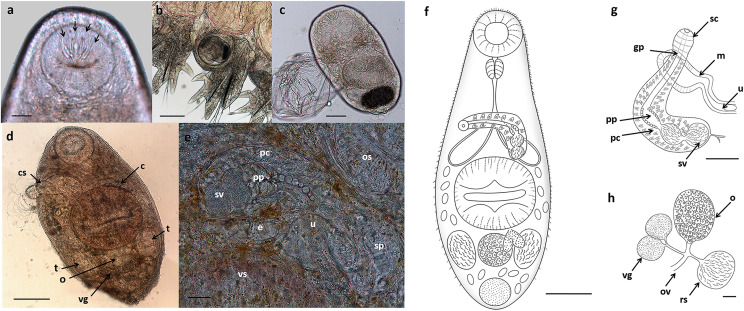
Microphotographs *in vivo* (a–e) and line drawings (f–h) of *Diphterostomum flavum* n. sp*.* (Zoogonidae) parasitizing the fish *Pinguipes brasilianus* on the Patagonian coast, Argentina: (a) anterior end of cercaria from the gastropod *Buccinanops deformis*, ducts of penetration glands ending in the oral sucker (arrows), (b) metacercariae naturally encysted in a parapodium of the polychaete *Kinbergonuphis dorsalis*, (c) metacercariae excysted at laboratory condition, (d) adult from the fish *Pinguipes brasilianus* with everted cirrus, (e) cirrus sac, (f) adult ventral view, (g) cirrus sac, (h) female genitalia. *Abbreviations*: c, caeca; cs, cirrus sac; e, egg; gp, genital pore; m, metraterm; o, ovary; ov, oviduct; os, oral sucker; pc, prostatic cells; pp, pars prostatica; rs, seminal receptacle; sc, spinous cirrus; sv, seminal vesicle; t, testis; u, uterus; vg, vitelline glands; vs, vental sucker. Scales: 20 μm (a, e, h), 50 μm (c, g), 100 μm (b, d, f).

**Figure 4:**
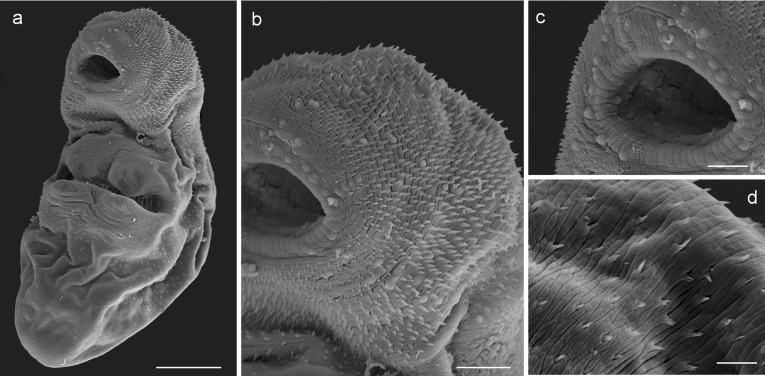
Scanning electron microscope (SEM) photographs of the adult stage of *Diphterostomum flavum* n. sp*.* (Zoogonidae) from the Patagonian coast, Argentina. (a) General ventral view. (b) Anterior part of body showing spines and papillae on ventral side. (c) Oral sucker showing papillae. (d) Detail of spines on ventral posterior part of body. Scales: 100 μm (a), 50 μm (b), 25 μm (c), 10 μm (d).

**Description:** (Figs. 3d–3h–3h and 4). Body elongated and oval, 710 (525–991) long by 222 (160–303) wide, yellow when alive. Posterior region slightly wider and more globular than anterior region. Yellow pigments densely distributed in parenchyma, mainly in older specimens. Tegument densely spiny in forebody; spines become more scattered and smaller toward posterior end (Fig. 4a). Numerous papillae in anterior region (ventral) and surrounding oral sucker (Figs. 4b and 4c). Spines tiny and bifurcated in hindbody (Fig. 4d). Oral sucker terminal, 109 (89–124) in diameter; prepharynx short, 7 (5–10) long; pharynx globular and muscular, 39 (28–54) long by 44 (35–57) wide; esophagus long and straight, 102 (54–142) long, divides into two saccular intestinal caeca, 134 (88–209) long by 49 (34–73) wide, usually terminating anterior to ventral sucker, sometimes slightly overlapping anterior end of ventral sucker. Ventral sucker orange or red, 188 (118–283) in diameter, two pairs of lip-like marginal lobes; sucker ratio 1:1.72 (1:1.32–1:2.28). Forebody 335 (231–465). Two testes equal and symmetrical, oval to cylindrical, in region of ventral sucker; left testis 104 (92–112) long by 69 (64–82) wide, right testis 93 (83–115) long by 75 (67–95) wide. Cirrus sac large, anterior to ventral sucker, elongated or recurved, containing bipartite seminal vesicle, prostatic pars and long cirrus, 224 (183–285) long by 54 (44–79) wide. Canalicular seminal receptacle posterior to ovary, 64 (56–73) long by 47 (45–51) wide. Genital pore on dorsal, on either left or right margin of body. Ovary dorsal, inter-testicular or slightly pre-testicular close to right testis, 89 (76–112) long by 64 (53–80) wide. Ootype and distal portion of Laurer’s canal not seen. Vitelline glands two compacted lobes at testes level, 47 (35–55) long by 35 (33–38) wide. Uterine loops filled with eggs from ventral sucker level to posterior end, never exceed ventral sucker. Eggs elliptical, large, embryonated, 31 (24–41) long by 14 (11–19) wide, membranous capsule. Excretory vesicle spherical, 70 (68–72) long by 74 (74–76) width.

#### Sporocyst, cercaria, and metacercaria

Although sporocysts with cercariae and metacercariae of *B*. *deformis* were described by Durio and Manter [24], here we provide new information about their morphology. **Sporocysts** (motionless, yellowish and with an elongated body) contain germinal balls, cercariae and/or encysted metacercariae. The majority of sporocysts (95%, *n* = 55) contain germinal balls and cercariae in different developing stages. The number of germinal balls and cercariae per sporocyst is 4 (1–10) and 6 (2–15), respectively. In the few sporocysts (5%, *n* = 3) where metacercariae were found, the number of metacercariae per sporocyst is 2 (1–5).

The **cercaria** is microcercous (tailless and with a tiny stylet present in the oral sucker), without eyespots, and bears a prominent ventral sucker with characteristic muscular lips. It possesses short saccular intestinal caeca and at least nine pairs of penetration glands separated in three groups; two groups with three and five pairs of glands opening ventrally at the anterior edge of oral sucker and one pair opening ventrally at the side of oral sucker (Fig. 3a). Developed cercaria has an undeveloped ovary at the left side of the body, a developing testis on each side of the body and an incompletely developed cirrus sac located between the intestinal caeca. The genital pore opens at the left side of the body at the pharyngeal level.

**Metacercaria encysted inside sporocyst**, spherical, 163 (157–168) diameter with thin wall 6 (5–7) thick. **Metacercaria encysted in polychaete** (Fig. 3b) similar, 176 (160–233) diameter and wall 6 (4–7) thick. Excysted metacercaria presents same structures as cercaria but lacks group of three penetration glands opening at anterior edge of oral sucker (Fig. 3c). Body 308 (254–349) long by 135 (92–164) wide; oral sucker 68 (64–71) long by 75 (59–85) wide, ventral sucker 95 (84–103) long by 101 (73–117) wide, and excretory vesicle 79 (62–95) long by 47 (35–53) wide.

##### Molecular data

The sequence of the amplified ITS2 fragment of adults of *D. brusinae* from *P. brasilianus* showed a single product of length 476 bp. After the sequence analysis, putative 5.8S and 28S regions were identified through comparisons with identical regions of other digeneans and found to be 73 and 98 nucleotides long, respectively. The sequence encoding for the ITS2 region presented 305 bp. No identical sequence was found in GenBank. The sequences encoding the ITS2 region of sporocysts from *B. deformis* and metacercariae from *K. dorsalis* were identical to the adult sequence.

The sequence of the amplified ITS2 fragment of *D. brusinae* from Portugal showed a single product of length 505 bp. Putative 5.8S and 28S regions presented 121 and 125 bp respectively and the ITS2 region presented 230 bp. The complete fragments (partial 5.8S-ITS2-partial 28S) of *D. brusinae* from Patagonia and Portugal were compared and these presented differences in 32 bp (distributed all along the sequence) and two gaps.

**Figure 5:**
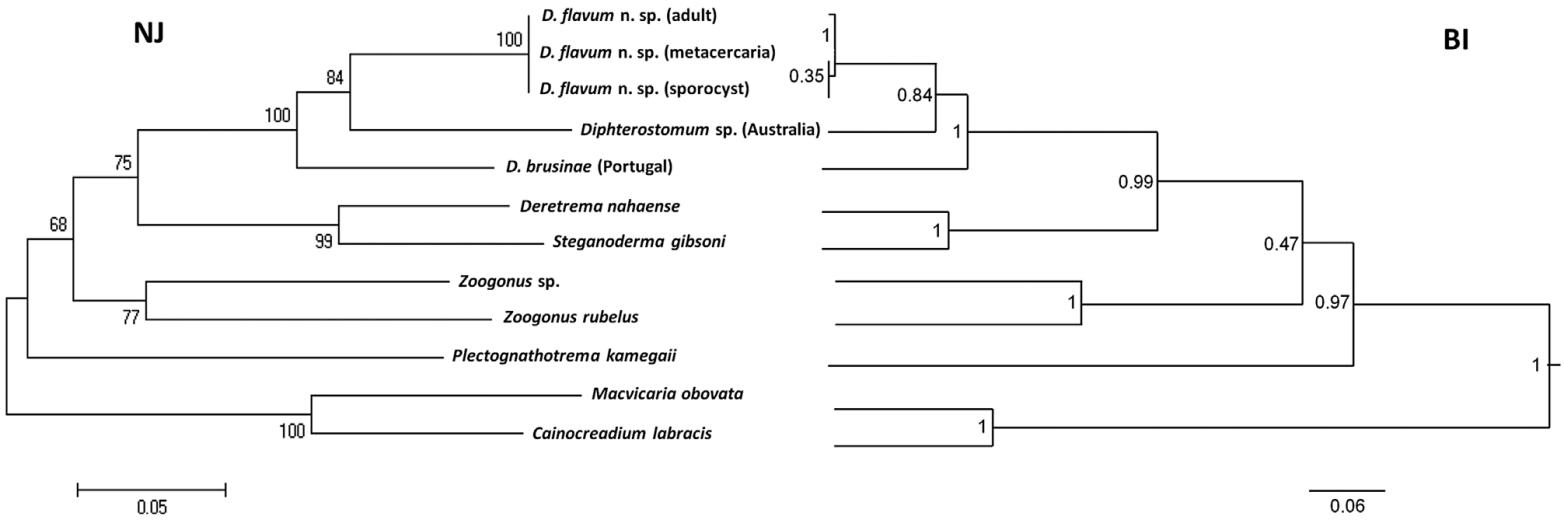
Clustering diagram depicting sequence similarity relationship among Zoogonidae species inferred from ITS2 rDNA. The tree was constructed using the neighbor-joining (NJ) method with pairwise deletion of gaps and Bayesian Inference (BI). NJ nodal numbers represent bootstrap values (%, *n* = 1000 replicates) and BI nodal support is indicated as posterior probabilities. Scale bar indicates substitutions/site.

Neighbor-joining (NJ) and Bayesian inference (BI) analyses resulted in trees with the same topology (Fig. 5). Moreover, both analyses revealed the presence of one clade for the genus *Diphterostomum* (posterior probability BI: 0.99; bootstrap NJ: 92%). *Diphterostomum flavum* n. sp. and *D. brusinae* present the lowest genetic distance (0.135) (Table 2). The genetic distance is a little higher between *D. flavum* n. sp. and *Diphterostomum* sp. (0.138). Genetic distances with species belonging to other genera were higher than 0.250.

**Table 2:** Pairwise nucleotide sequence comparisons between zoogonid species calculated as the percentage of nucleotide differences (gaps treated as missing data) for the aligned ITS2 sequences (*n* = 403 bp).

	1	2	3	4	5	6	7	8
1. *Diphterostomum flavum* n. sp.								
2. *Diphterostomum brusinae*	0.135							
3. *Diphterostomum* sp.	0.138	0.172						
4. *Deretrema nahaense*	0.250	0.241	0.278					
5. *Lecithostaphylus gibsoni*	0.286	0.256	0.288	0.127				
6. *Plectognathotrema kamegaii*	0.296	0.287	0.325	0.318	0.314			
7. *Zoogonus* sp.	0.295	0.301	0.301	0.288	0.275	0.269		
8. *Zoogonus rubellus*	0.327	0.274	0.314	0.262	0.277	0.314	0.219	

##### Taxonomic remarks

The zoogonid adult described here is morphologically indistinguishable from *Diphterostomum brusinae* first described by Stossich [74]. As Bray [9] pointed out, this species is reported mostly from the Mediterranean and Black Seas, but is also recorded in a wide variety of sites in the Atlantic, Indian, and Pacific Oceans (Fig. 1). At least 34 records of *D. brusinae* exist around the world, including for six species which were later synonymized (Fig. 1). This species is characterized by having two pairs of lip-like marginal lobes on the ventral sucker, a large cirrus sac anterior to ventral sucker containing a bipartite seminal vesicle and well developed prostatic complex, genital pore sinistral, intertesticular ovary, vitellarium as two compact masses and a uterus filled with large and elliptical eggs [10, 81]. Pina et al. [60] compared some measurements of adults described by Stossich [74], Looss [39], Stafford [73], and Palombi [54, 55] and did not find significant differences. Adults here described have measurements in agreement with these previous works. The sole morphological difference found is in the number of penetration glands of the cercaria. Palombi [54] described two groups without specification of gland number and Pina et al. [60] described three pairs of penetration glands. Cercariae described by Gilardoni et al. [31] were erroneously characterized as having three pairs of penetration glands. New exhaustive morphological study of cercariae allowed recognition of at least nine pairs of penetration glands. Additionally, molecular analysis of ITS2 sequences support the differences between *Diphterostomum flavum* n. sp., *D. brusinae* from Portugal and *Diphterostomum* sp. from Australia (see Molecular data section and Fig. 4). In the Argentinean Sea, three species of *Diphterostomum* have been recorded: *Diphterostomum americanum* Manter, 1947 from Puerto Quequén [43], *Diphterostomum* sp. from North Patagonia [15], and *D. brusinae* from Buenos Aires Province and North Patagonia [43, 77]; the last of these is identified here as *D. flavum* n. sp.

##### Host spectrum and experimental infection

All the dissections seeking natural infections in the clam (*A. petitiana)*, the crab (*O. trimaculatus)*, and the polychaetes (see species in Table 1) were negative. In the experimental infections, the clam *A. petitiana* was never infected, but the polychaetes from Puerto Madryn were successfully infected. The prevalence of metacercariae in the experimentally infected *Boccardia proboscidea* was 84% (*n* = 20) and the mean intensity was 5.76 (1–17), and in *Platynereis* sp. was 75% (*n* = 4) and the mean intensity was 8.33 (3–14). Both overall prevalence (83.33% vs. 22.22%) and mean intensity (6.15 (1–17) vs. 1.68 (1–3)) were significantly higher in experimentally than naturally infected polychaetes *K. dorsalis* (*Z* = −4.64, *p* < 0.05; *Z* = −7.48, *p* < 0.05). The measurements of metacercariae experimentally encysted in polychaetes are similar to those from natural infections: cyst 172 (159–190) diameter, wall 5 (3–9) thick, body 316 (228–379) long by 123 (86–159) wide, oral sucker 61 (48–74) long by 66 (54–78) wide, ventral sucker 83 (66–99) long by 91 (80–118) wide, and excretory vesicle 63 (46–82) long by 58 (40–73) wide.

## Discussion

This work describes a new species of *Diphterostomum*, named *Diphterostomum flavum* n. sp., and its life cycle both naturally and experimentally elucidated by morphological and molecular data. From this study, the existence of cryptic species concealed among forms identified as *Diphterostomum brusinae* is based on three lines of evidence: (1) a strong morphological character from the cercaria as the number of penetration glands [31], (2) the strict host specificity to the first intermediate host (Fig. 2) [36] and host ecological factors such as feeding habits of the definitive hosts [1], and (3) genetic differences in the ITS2 sequences and genetic distance between species [19].

First, the adult form of *D. brusinae* is characterized by having conspicuous lips on its ventral sucker and, to date, this has been the main diagnostic character considered to recognize the species. Several species that had been described as different from *D. brusinae* in the past were later synonymized with *D. brusinae* (see Fig. 1) because they lack conspicuous morphological differences and shared the feature of having four lips on the ventral sucker. Because of the lack of conspicuous morphological differences, trematodes have the highest reported level of cryptic diversity [61]. Specifically, most trematodes lack hard parts, especially in their terminal genitalia, which can be of great assistance in species differentiation [61]. Larval stages can be difficult to distinguish morphologically between species [38, 45]; however, some diagnostic characters of cercariae can be useful including stylet shape, size of suckers, shape of excretory vesicle, and number of penetration glands (e.g., [6, 31, 32]). For *D. brusinae*, Palombi [54] described two groups of penetration glands in Italian cercariae, Pina et al. [60] described three pairs of glands in Portuguese cercariae, and at least nine pairs of glands separated into three groups are recorded in this study in cercariae from the Southwestern Atlantic Ocean.

Secondly, several studies have demonstrated that the free-swimming miracidial stage that infects the molluscan host shows a strict specificity (e.g., [28, 30, 36, 52, 75]) with the phylogenetic relationships of the hosts driving this speciﬁcity [2, 22]. At the species level, digenean intra-molluscan stages are generally only capable of successful development within a circumscribed set of hosts and it is unusual for a digenean species to complete development in molluscs from more than one family [2]. Some studies have considered sympatric and phylogenetically related snail species. For example, two pulmonate limpets, *Siphonaria lessonii* Blainville and *S. lateralis* Gould (Siphonariidae), share a parasite species, the microphallid *Maritrema madrynense* Diaz & Cremonte, 2010; these two limpets are ecologically and genetically very similar and can be found together in the same intertidal region from South Patagonia, Argentina [20]. At the same site, *Crepipatella dilatata* (Lamarck) (Calyptraeidae) inhabits the lower intertidal and subtidal zone and is infected by larvae of a microphallid species very similar to *M. madrynense* but possessing a different stylet and number of penetration glands [31, 32]. Here it is evident that the specificity for the first intermediate host is related to parasite-host co-evolution and morphological differences between larvae.

The cercaria of *D. brusinae* has been recorded in four nassariid and one naticid gastropod species in the Mediterranean, Black Sea, and North of Portugal [21, 54, 55, 60]. Given the geographic distances involved and the overall pattern of host specificity, it is probable that further species of *Diphterostomum* are present in Europe and on the other continents where it has been recorded.

The degree of specificity of trematode metacercariae to second intermediate hosts is usually lower than that to first intermediate or definitive hosts. The use of a variety of hosts for trophic transmission, increases their chances of being transmitted to the definitive host [33]. Our experimental infections were successful for polychaetes but unsuccessful for a bivalve as second intermediate hosts. Some bivalves such as *Chamelea gallina* (Linnaeus) (Veneridae), *Spisula subtruncata* da Costa (Mactridae), and *Cerastoderma edule* Linnaeus (Cardiidae) have been recorded as second intermediate hosts for *D. brusinae* from the Black Sea and Portugal on the Northeastern Atlantic coast [21, 60]. In addition, Martorelli et al. [43] experimentally infected the bivalve *Limnoperna fortunei* (Dunker) (Mytilidae) with cercariae of *Diphterostomum brusinae* from *B. deformis*. In our study, the most common bivalve *Ardeamya petitiana*, which co-habits with the first intermediate host, *B*. *deformis*, was never found naturally parasitized. In addition, all attempts at experimental infection failed. This incompatibility could be explained by the feeding habit of *A. petitiana* which is a deposit feeder that does not expose the mantle when feeding; thus, cercariae cannot enter. Cremonte [17] demonstrated that a gymnophallid cercaria can enter the bivalve *Darina solenoides* (King) (Mactricidae), but never *A. petitiana* because the former species exposes the mantle border when feeding, allowing the larvae to penetrate.

The molecular differences among *Diphterostomum* species clearly support the existence of cryptic species. Vilas et al. [78] suggest that a greater than 1% difference with ITS markers indicates separate species for trematode parasites. The genetic distance between *D. flavum* n. sp. and *D. brusinae* from Portugal was 13.1% and the genetic distance between *D. flavum* n. sp. and *Diphterostomum* sp. from Australia was 12.6%. Despite the limited available sequences of *Diphterostomum* species, the molecular differentiation is clear and allows species delineation. The rest of the phylogenetic relationships did not agree with others performed with the 28S region [71]. Our conclusions are limited to distinguishing among *Diphterostomum* species, because the ITS2 marker is adequate for this purpose; however, it is not a robust marker for deep-level phylogenetic inference.

In conclusion, the combination of morphology, ecology, and genetics suggests strongly the existence of cryptic species otherwise identifiable as *D. brusinae*, which has been widely recorded around the world. Although molecular analyses are a powerful tool to discriminate among cryptic species, knowledge of digenean life cycles, and the ecology of their hosts adds important biological context to the delineation of such species.
